# Evaluation of knowledge, impacts and government intervention strategies during the COVID – 19 pandemic in Nigeria

**DOI:** 10.1016/j.dib.2020.106177

**Published:** 2020-08-17

**Authors:** Funmilayo V. Doherty, Olumide A. Odeyemi, Abdullahi Adeola, Oluwatosin Amolegbe, Folashade Eyitayo Ajagbe

**Affiliations:** a Environmental Biology Unit, Department of Biological Science, Yaba College of Technology, Lagos, Nigeria; b Office of Research Services, Research Division, University of Tasmania, Launceston, TAS, Australia

**Keywords:** SARS-CoV-2, Intervention strategies, COVID-19, Public health

## Abstract

The SARS-CoV-2 is a novel strain of coronavirus which is ravaging many countries, and this has become a global public health concern. With the increasing number of COVID-19 confirmed cases and deaths in Nigeria, the pandemic has led to massive public reactions. This data attempted to evaluate the knowledge, impacts, and government intervention during the pandemic. An online survey was conducted using a questionnaire shared via social media using a Snowball sampling technique. The data were analyzed using descriptive statistics and analysis of variance (ANOVA). A total of 387 responses was received. Results show that a significant number of respondents had adequate knowledge about COVID-19 modes of transmission, symptoms, and preventive measures. Respondents maintain personal hygiene as 67% wash their hands with soap. The pandemic has caused worry (65%), anxiety (42%), panic (35%), and depression (16%) among respondents, even as government intervention is seen as inadequate by 70%. There is a need for mental health support and increased information campaigns about COVID-19.

**Specifications Table**

**Table:** 

Subject	Global public health
Specific subject area	Knowledge, impacts and intervention strategies towards COVID-19
Type of data	Primary data collected via online survey
How data were acquired	Primary data collected via online survey (Google form) and statistically analysed using SPSS. Supplementary survey questionnaire provided.
Data format	The raw data was collected as responses in Google spreadsheets, exported to SPSS for analysis after filtering to remove uncompleted responses.
Parameters for data collection	A total of 387 Nigerians (aged from16 years to 61 years) were interviewed on their perception of the COVID-19 pandemic.
Description of data collection	An online survey was conducted using a questionnaire shared via social media using a Snowball sampling technique.
Data source location	The participants were residents in different parts of Nigeria however, the majority of about 250 respondents were residents in Lagos which is the epicenter of COVID-19 in Nigeria, 33 respondents were residents in Ogun state and 13 respondents were residents in the Federal Capital Territory. The remaining 91 respondents were from 23 states in the middle belt, northern, southwest, and south-eastern parts of Nigeria.
Data accessibility	Repository name: Mendeley Data identification number: 10.17632/532zhmsxzk.1 Direct URL to data: https://data.mendeley.com/datasets/532zhmsxzk/1

**Value of the Data**

• With the increasing number of COVID-19 confirmed cases and deaths in Nigeria, the pandemic has led to massive public reactions. This data attempted to evaluate the knowledge, impacts, and government intervention during the pandemic.
• This data could be used by relevant authorities to reinforce their strategies in mental health support to buffer against COVID-19 pandemic-related stress.
• The data can be analysed and compared with existing or new datasets.
• The dataset will be useful to improve government intervention strategies
• The data would also be useful for mathematical modeling, particularly in the inclusion of individual responses-besides the large uncertainties associated with the transmission of a novel, emerging pathogen.

## 1 Data description

### 1.1 Part A: socio-demographic information of respondents

The data was conducted among 387 respondents, from March 27th – April 7th, 2020. The participants were residents in different parts of Nigeria however, the majority of respondents (250) were from Lagos which is the epicenter of COVID-19 in Nigeria, 33 respondents were residents in Ogun state and 13 respondents were residents in the Federal Capital Territory. The remaining 91 respondents were from 23 states in the middle belt, northern, southwest, and southeastern parts of Nigeria (Table 1).

**Table 1:** Socio-demographic information of the respondents.

Characteristics		N	F (%)
Age			
	16 - 21	38	10
	21 - 30	168	43
	31 - 40	93	24
	41 - 50	56	15
	51 - 60	26	7
	61 - above	6	2
Gender			
	Female	225	58
	Male	162	42
Education			
	Secondary school	5	1
	National diploma	28	7
	HND/BSc	217	56
	Post-graduate (PGD, MSc, Ph.D.)	137	35
Marital status			
	Single	198	51
	Engaged	11	3
	Married	171	44
	Separated	4	1
	Divorced	3	1
Employment status			
	Employed	185	48
	Unemployed	116	30
	Self employed	86	22

Where *n* = number of respondents, *f* = frequency in percentages.

### 1.2 Part B: level of awareness about COVID-19 pandemic

All the respondents have heard about the COVID-19 pandemic. As shown in Table 2, 85% of the responders know about the mode of transmission, symptoms, and preventive measure of the disease; 55% of the respondents are aware of the causative agent of COVID-19. Many of the respondents (74%) heard about COVID-19, through the mass media and over a month ago (as at the time of the survey). The perceived sadness and fear for COVID-19 were high among the respondents as 70% stated that they are afraid of hearing the news about the virus. A significant number of participants (92%) acknowledged that their communities are aware of COVID-19 through mass media and faith-based organizations. Most participants (94%) indicated that social distancing can help prevent the spread of the virus.

**Table 2:** Awareness of COVID-19.

Awareness	Responses (*N* = 387)				
Have you heard about COVID-19?	**Yes**	**No**			
N	386	1			
F	99.7	0.3			
What aspects of the COVID-19 do you know about?	**Cause**	**Transmission**	**Symptoms**	**Preventive measures**	
Yes	215	332	355	344	
F	55.6	85.8	91.7	88.9	
No	172	55	32	43	
F	44.4	14.2	8.3	11.1	
How did you hear about COVID - 19?	**Mass media**	**Social media**	**Friends**	**Colleagues**	**Church/Mosques**
Yes	287	338	135	119	90
F	74.2	87.3	34.9	30.7	23.3
No	100	49	252	268	297
F	25.8	12.7	65.1	69.3	76.7
When did you hear about it?	**This week**	**A week ago**	**A month ago**	**Over a month ago**	
N	2	20	91	274	
F	0.5	5.2	23.5	70.8	
Are you afraid/sad from hearing news about the virus?	**Yes**	**No**	**Maybe**		
N	273	70	44		
F	70.5	18.1	11.4		
Do you feel your community has heard about COVID-19?	**Mass media**	**Social media**	**Churches/Mosques**		
Yes	294	279	93		
F	76.0	72.1	24.0		
No	93	108	294		
F	24.0	27.9	76.0		
Social distancing can help prevent the spread of the virus?	**Yes**	**No**	**Not sure**		
N	363	5	19		
F	93.8	1.3	4.9		

Where *N* = total number of respondents and *f* = frequency (%.

### 1.3 Part C: respiratory and personal hygiene

Data in Table 3 shows there is a perceived capability to avoid COVID-19 among the subjects. Substantial proportions (52%) of respondents reported that they do cover their mouth with a tissue or handkerchief/elbow when sneezing or coughing as always recommended, 33% of respondents indicated most of the time,13% indicated occasionally and 2% do not cover their mouth. There is a similar attitude of the respondents toward the use of alcohol-based hand rub or soap hand-wash when washing their hands, as 67% wash their hands with soap. Only 30% of the respondents do not wear any of the personal protective equipment (PPE) as shown in the Table. About 70% either wear PPE always, most of the time according to risk assessment or occasionally. Four percent of the participants reported having had close contact (within 1 meter) with a suspected or confirmed victim of COVID-19 while 88% of them believed they had not as shown in Table 3. Three-quarters of the respondents reported that they sanitize/wash their hands after using an automated teller machine (ATM/POS); while those that sanitize/wash their hands after touching Nigerian currency were 59%. Majority of the respondents (over 90%) acknowledged that staying at home will help reduce the spread of COVID −19. Little above half of the respondents choose social distancing, movement restriction and use of face mask as precautionary measures they take against COVID-19 infection as shown in Table 3.

**Table 3:** Respiratory and personal hygiene.

Respiratory and personal hygiene	Responses (*N* = 387)			
Do you cover your mouth with a tissue or handkerchief / elbow when sneezing or coughing?	**Always as recommended**	**Most of the time**	**Occasionally**	**Not at all**
N	202	128	49	8
F	52.2	33.1	12.7	2.1
Do you use alcohol-based hand rub or soap hand-wash when washing your hands?	261	99	21	6
F	67.4	25.6	5.4	1.6
Do you wear any of the following PPE when necessary ? (PPE includes: Face mask, Face shield, Gloves, Head cover)	**Always as recommended**	**Most of the time**	**Occasionally**	**Not at all**
N	99	72	99	117
F	25.6	18.6	25.6	30.2
Have you had close contact (within 1 meter) with a suspected or confirmed victim of COVID-19?	**Yes**	**No**	**Maybe**	
N	16	343	28	
F	4.1	88.6	7.2	
Do you sanitize/wash your hands after using the ATM/POS machine?	**Yes**	**No**	**Can't remember**	
N	296	49	42	
F	76.5	12.7	10.9	
Do you sanitize/wash your hands after touching naira notes?	230	125	32	
F	59.4	32.3	8.3	
Do you believe the stay at home order will help reduce the spread of Coronavirus?	**Yes**	**No**	**Maybe**	
N	351	10	26	
F	90.7	2.6	6.7	
What precautionary measures do you take against COVID-19?	**Social distancing**	**Movement restriction**	**Use of face mask**	**All of the above**
No	25	35	2	209
F	6.5	9.0	0.5	54.0

Where *n* = number of respondents, *f* = frequency in percentages and *N* = total number of the respondents.

### 1.4 Part D: impacts of COVID-19

About 68% of the respondents pointed out that their personal or family's lifestyle is affected by the outbreak of COVID-19 while one-quarter said that it does not affect them as shown in Table 4. Similar responses were also recorded on the effect of precautionary measures on their ability to do their job as well as financial losses. A substantial number of the participants expressed emotional distress, as evidenced by worry (65%), anxiety (42%), panic (35%), depression (16%), while 3% are happy with the COVID-19 pandemic. During the stay at home order, 78% of the respondents indicated that they make use of the phone, two-third were spending time with their families, about half of the respondents were engaged in book reading, listening to music, while nearly three-quarter were sleeping, praying and resting. Thirty percent of respondents were engaged in exercises as shown in Table 4. The confirmation of cases of COVID-19 in Nigeria and the public perception of the disease and concerns about Nigeria's level of preparedness are already fueling fear and panic in the country.

**Table 4:** Impacts of COVID-19.

Impacts of COVID-19	Responses (*N* = 387)							
Is your personal or family's lifestyle affected with the outbreak of COVID-19?	**Yes**	**No**	**Maybe**					
N	265	99	23					
F	68.5	25.6	5.9					
Do the precautionary measures affect your ability to do your job?	264	100	23					
F	68.2	25.8	5.9					
Are the precautionary measures affecting your income?	245	104	38					
F	63.3	26.9	9.8					
Has the coronavirus pandemic affected your emotions?	**Anxiety**	**Worry**	**Panic**	**Depression**	**Happy**	**Indifferent**		
Yes	165	252	139	64	15	95		
F	42.6	65.1	35.9	16.5	3.9	24.5		
No	222	135	248	323	372	292		
F	57.4	34.9	64.1	83.5	96.1	75.5		
Which of the following do you do during the stay at home order?	**Read a book**	**Listen to music**	**Spend time with family**	**Exercise**	**Sleep and rest**	**Use your phone**	**Work at home**	**Pray**
Yes	220	191	261	118	284	305	165	269
F	56.8	49.4	67.4	30.5	73.4	78.8	42.6	69.5
No	167	196	126	269	102	82	222	118
F	43.2	50.6	32.6	69.5	26.4	21.2	57.4	30.5

Where *n* = number of respondents, *f* = frequency in percentages and *N* = total number of the respondents.

### 1.5 Part E: government intervention

The Nigeria government had reinforced its level of preparedness through public sensitization, the establishment of in-country COVID-19 diagnostic laboratories and isolation facilities, enforcement of lockdown order, and provision of palliatives to citizens. However, the survey, as shown in Table 5 indicated that the government's intervention in managing and curbing the spread of COVID-19 was insufficient as 59% of respondents believed that government intervention was insufficient. Meanwhile, about 3/4th of the participants reported that the provision of personal protective equipment (PPEs) was insufficient, while 11% answered that there was no provision of PPEs. Moreover, it shows that only 18% believed that the government had provided adequate PPEs for her health workers in combating COVID-19, 37% of respondents disagreed while 44% were not sure. As shown in Table 5, over 30% of the respondents either agreed or disagreed that the information provided by the health authorities to the public was accurate, while 27% of respondents remained indifferent. Similar opinions were recorded for the action taken by the health authority in each state of residence of the participants. Table 5 shows that equal proportions (about one fourth) of the participants disagree, agree, or indifferent that they have had the chance to express their personal views and concerns to the authorities if they wanted to. To alleviate the burden caused by the lockdown effect of COVID-19 on the citizens, over 70% of the participants felt that relief materials and support provided by the government to her citizens were inadequate. In contrast, 10% agreed on the adequacy of materials and support provided by the government as shown in Table 5.

**Table 5:** Government intervention strategies.

Government intervention strategies	Responses (*N* = 387)			
What is your assessment of government's intervention in COVID-19 management by stopping the spread?	**Insufficient**	**Sufficient and timely**	**Sufficient and untimely**	**Not at all**
N	228	42	97	20
F	58.9	10.9	25.1	5.2
What is your assessment of government's intervention in COVID-19 management through providing adequate PPEs for the public?	281	36	27	43
F	72.6	9.3	7.0	11.1
With regards to the distribution of information by the health authorities to the public, do you agree or disagree that it has generally been accurate?	**Strongly disagree**	**Disagree**	**Neutral**	**Agree**
n	53	80	108	121
f	13.7	20.7	27.9	31.3
With regard to the distribution of information by the health authorities to the public in your state of residence, do you agree or disagree that it has generally been sufficient?	50	89	99	120
f	12.9	23.0	25.6	31.0
Do you agree or disagree that you have had the chance to express your personal views and concerns to the authorities if you wanted to?	57	104	98	108
f	14.73	26.87	25.32	27.91
The government is providing adequate relief materials and support for her Citizens due to the total lock down brought by COVID-19	165	111	65	41
f	42.64	28.68	16.80	10.59
The government is providing adequate PPE for her health workers in combating COVID-19	**Yes**	**No**	**Maybe**	
N	73	143	171	
F	18.9	37.0	44.2	

Where *n* = number of respondents, *f* = frequency in percentages and *N* = total number of the respondents.

It was observed as shown in Table 6 that the knowledge of the outbreak of COVID-19 was influenced by the age (*p* < 0.05), gender (*p* < 0.05), level of education (*p* < 0.05), marital status (*p* < 0.05) and employment status (*p* < 0.05).. Both age (*p* = 0.03) and level of education (*p* < 0.05) influenced the respiratory and personal hygiene of the respondents while only age significantly (*p* = 0.02) influenced the perception of the respondents towards the government intervention strategies. None of the socio-demographic variables significantly (*p* > 0.05) influenced the perception of the respondents on the impact of the outbreak and lockdown. The data showed that 99.7% of the respondents were aware of the outbreak irrespective of age, gender, level of education, marital and employment status and the participants were all affected although at different levels.

**Table 6:** Effect of socio-economic information on awareness, personal hygiene, impact, and government intervention strategies.

Variables		Knowledge	Myths	Personal hygiene	Impact	Government intervention
**Age**						
	Sum of Squares	174.61	116.58	101.53	52.40	191.47
	Df	5	5	5	5	5
	Mean Square	34.92	23.32	20.31	10.48	38.29
	F	4.16	1.92	2.52	1.27	2.86
	Sig.	0.00*	0.09	0.03*	0.28	0.02*
**Gender**						
	Sum of Squares	178.60	61.33	30.32	0.57	13.13
	Df	1	1	1	1	1
	Mean Square	178.60	61.33	30.32	0.57	13.13
	F	21.51	5.05	3.72	0.07	0.96
	Sig.	0.00*	0.03*	0.06	0.79	0.33
**Level of Education**						
	Sum of Squares	227.89	58.48	135.38	39.68	35.36
	Df	3	3	3	3	3
	Mean Square	75.96	19.49	45.13	13.23	11.79
	F	9.24	1.60	5.69	1.60	0.86
		0.00*	0.19	0.00*	0.19	0.46
**Marital status**						
	Sum of Squares	175.73	115.96	44.27	25.50	94.49
	Df	4	4	4	4	4
	Mean Square	43.93	28.99	11.07	6.38	23.62
	F	5.25	2.40	1.35	0.77	1.73
	Sig.	0.00*	0.05	0.25	0.55	0.14
**Employment status**						
	Sum of Squares	204.66	17.63	42.42	22.43	29.66
	Df	2	2	2	2	2
	Mean Square	102.33	8.82	21.21	11.22	14.83
	F	12.39	0.72	2.60	1.35	1.08
	Sig.	0.00*	0.49	0.08	0.26	0.34

⁎ *p* < 0.05 (significant).

## 2 Experimental design, materials and methods

This was a cross-sectional data carried out in Nigeria and a Snowball sampling technique was used. Using the Google form, an online questionnaire was developed. The questionnaire was distributed through electronic means, and this was easily shared on social media including WhatsApp [1] which is a smartphone/desktop enabled social media platform with over one billion global users [2]. The participants who were the first point of contact were encouraged to disseminate the link of the survey to other contacts, this enabled the survey to reach as many respondents as possible. Data collected through the Google form commenced from March 27 to April 7, 2020, during the lockdown period in Nigeria.

Participation in the data was voluntary and participants could withdraw at any time. On receiving and clicking the link, participants were auto directed to the synopsis that explained the purpose of the data, and only the participants that consented were included in the data. Participants were directed to complete the demographic details then a set of several questions followed which the participants were to answer. No personal or private data was collected and similarly, the data collection procedure followed the provisions of the Declaration of Helsinki on human subjects [3].

It was an online data therefore participants with access to the internet could participate. All participants resident in any of the states in Nigeria, 16 years and above, were eligible. The authors were able to collect data from across various states of Nigeria. The socio-demographic variables included age, gender, employment status, education, marital status, and state of residence.

The questionnaire (Part A – E) consisted of the following sections: socio-demographic information as described by Odeyemi et al. [4]., awareness of COVID-19, respiratory and personal hygiene undertaken by participants, impacts on health, emotion, finances, and the effectiveness of government intervention. Questions on the awareness of COVID-19 that were asked included symptoms of the disease, source (s) of information about the outbreak, time of awareness, and social distancing. Eight questions on respiratory and personal hygiene such as the covering of the mouth with a tissue or handkerchief/elbow when sneezing or coughing, the use of soap or alcohol to sanitize the hands, wearing personal protective equipment (PPE), social distancing, washing or sanitizing hands after handling / withdrawing cash, and effect of the lockdown. The researchers also examined the impact of the outbreak on the participants’ personal or family lifestyle, job, income, and emotions. Similarly, the survey asked the participants if they spent time with family and or did the following: reading books, listening to music, exercising, sleeping/resting, using phone, praying, and working remotely. The last part evaluated the perception of the participants regarding the government's intervention strategies in curbing the spread of the virus, adequate provision of PPE to the health workers and citizens [5], timely dissemination of COVID-19 related information to the public and provision of relief materials.

The above questions were then designed and grouped into six parts (A-E) using Google form. Google form was used because it enabled the questionnaire to be sent electronically while the responses are collected in real-time and automatically available in Google spreadsheets. More so, due to the lockdown, the questionnaire could not be distributed physically. Therefore, there was no facility to check the validity of the information provided by the respondents thereby making it impossible to track biases. Nevertheless, this is not without agreement with previous findings on the impacts of outbreak of virus/disease on public's emotion and behavior [6], [7]

The data obtained were analyzed using descriptive statistics to describe the socio-demographic information [8], while analysis of variance (ANOVA) was used to describe the effect of the independent variables on awareness, myths, impacts and government intervention using statistical package for social sciences – IBM SPSS v 25 [6,8].

## Declaration of Competing Interest

The authors declare that they have no known competing financial interests or personal relationships which have, or could be perceived to have, influenced the work reported in this article.

## Appendix Supplementary materials

Image, application 1

Image, application 2
