# Supplementary material for: Evaluation of knowledge, impacts and government intervention strategies during the COVID – 19 pandemic in Nigeria
Source: Data Brief. 2020 Aug 17;32:106177. doi: 10.1016/j.dib.2020.106177 (PMC7430239; doi:10.1016/j.dib.2020.106177)
Supplement: Supplementary file 1 [file mmc1.docx]

**EVALUATION OF MYTHS, IMPACTS AND GOVERNMENT INTERVENTION TOWARDS COVID-19 AMONG NIGERIANS**

***Part A: Socio-demographic information***

1. **Age**
   1. 16 – 20
   2. 21 – 25
   3. 26 – 30
   4. 31 – 35
   5. 36 – 40
   6. 41 - above
2. **Gender**
   1. Male
   2. Female
3. **Education**
   1. No formal education
   2. National Diploma
   3. Higher National Diploma/BSc
   4. Postgraduate (PGD, MSc, PhD)
4. **Employment status**
   1. Employed
   2. Unemployed
   3. Self employed
5. **Marital status**
   1. Single
   2. Engaged
   3. Married
   4. Separated
   5. Divorced

**Part B: Awareness**

1. Have you heard about COVID-19?

Yes/No

2. If yes, how did you hear about it?

Mass media

Social media

Through friends

Colleagues

Churches/Mosques

3. When did you hear about it?

a. This week

b. A week ago

c. More than two weeks ago

4. Are you afraid/sad from hearing news about the virus?

Yes/No

4. Do you feel your community has heard about COVID-19?

Yes/No

**5.** If yes, through which of the following sources?

□ Mass media □ social media □ faith-based organizations such as church and mosques □ All the above

6. Social distancing can help prevent the spread of the virus?

Yes/No

**Part C: Myths**

1. COVID-19 virus cannot be transmitted in areas with hot climates or weathers

□ Agree □ Not agree □ Unknown

2. Taking hot drinks prevent the new coronavirus disease

□ Agree □ Not agree □ Unknown

3. COVID-19 can only be transmitted in humid weather?

□ Agree □ Not agree □ Unknown

4. Coronavirus can be transmitted through mosquito bites

□ Agree □ Not agree □ Unknown

5. Hot bath can prevent the new coronavirus disease

□ Agree □ Not agree □ Unknown

6. Eating spicy food/pepper soup can prevent the new coronavirus disease

□ Agree □ Not agree □ Unknown

7. Use of hand dryers can kill the new coronavirus

□ Agree □ Not agree □ Unknown

8. Ultraviolet (uv) disinfection lamp can kill the new coronavirus

□ Agree □ Not agree □ Unknown

9. rinsing nose with saline (salt) water can prevent infection with the new coronavirus

□ Agree □ Not agree □ Unknown

10. The new coronavirus can only affect older people

□ Agree □ Not agree □ Unknown

11. Chloroquine can cure Coronavirus

□ Agree □ Not agree □ Unknown

**Part D: Respiratory and personal hygiene**

1. Did you cover your mouth with a tissue or handkerchief / elbow when sneezing or coughing?

□ Always, as recommended

□ Most of the time

□ Occasionally

□ Not at all

2. Do you use alcohol-based hand rub or soap hand-wash when washing your hands in the past few weeks?

□ Always, as recommended

□ Most of the time

□ Occasionally

□ Not at all

1. Do you wear any of the following PPE when indicated?

(PPE includes: Face mask, Face shield, Gloves, Head cover)

□ Always, according to the risk assessment

□ Most of the time, according to the risk assessment

□ Occasionally

□ Not at all

4. Have you had close contact (within 1 meter) with a suspected or confirmed victim of COVID-19?

□ Yes □ No □ Unknown

1. Do you sanitize your hands after using the ATM?

Yes/No

1. Do you sanitize your hands after touching naira notes?

Yes/No

1. Do you believe the stay at home order will help reduce the spread of Coronavirus?

Yes/ No

1. What precautionary measures do you take against COVID-19?

Social distancing

Movement restriction

Use of face mask

All of the above

**Part E: Impacts**

1. Is your personal or family’s lifestyle affected with the outbreak of COVID-19?

□ Yes □ No □ Unknown

2. Is the precautionary measures affect your ability to do your job

□ Yes □ No □ Unknown

3. Is the precautionary measures affect your income

□ Yes □ No □ Unknown

4. Are you affected emotionally/ mentally by the negative reports/ news of Coronavirus globally?

Yes/No

5. Which of the following do you do during the stay at home order:

Read a book

Listen to music

Spend time with family

Exercise

Watch news

Spend time on your phone

Sleep and rest more

Work at home

Seek the face of God

**Part F: Government intervention**

1. What is your assessment of government’s intervention in COVID-19 management by stopping the spread?

□ Insufficient □ Sufficient and Timely □ Sufficient but untimely □ Not at all

2. What is your assessment of government’s intervention in COVID-19 management by providing adequate PPEs for the public?

□ Insufficient □ Sufficient and Timely □ Sufficient but untimely □ Not at all

3. With regards to the distribution of information by the health authorities to the public, do you agree or disagree that it has generally been accurate?

(a) Strongly disagree (b) Disagree (c) Not sure, but probably disagree (d) Not sure, but probably agree (e) Agree

(f) Strongly agree

4. With regards to the distribution of information by the health authorities to the public in your country, do you agree or disagree that it has generally been sufficient?

(a) Strongly disagree (b) Disagree (c) Not sure, but probably disagree (d) Not sure, but probably agree (e) Agree

(f) Strongly agree

5. Do you agree or disagree that you have had the chance to express your personal views and concerns to the authorities if you wanted to?

(a) Strongly disagree (b) Disagree (c) Not sure, but probably disagree (d) Not sure, but probably agree (e) Agree

(f) Strongly agree

6.The government is providing adequate Personal Protective Equipment for her health workers and the public in combating COVID-19

Strongly Agree Strongly Disagree, Unknown

7. The government is providing adequate relief materials and support for her Citizens due to the total lock down brought by COVID-19

Strongly Agree, Strongly Disagree, Unknown
